# Resource Availability and Spatial Heterogeneity Control Bacterial Community Response to Nutrient Enrichment in Lakes

**DOI:** 10.1371/journal.pone.0086991

**Published:** 2014-01-28

**Authors:** KathiJo Jankowski, Daniel E. Schindler, M. Claire Horner-Devine

**Affiliations:** University of Washington, School of Aquatic and Fisheries Sciences, Seattle, Washington, United States of America; Centro de Investigación y de Estudios Avanzados, Mexico

## Abstract

The diversity and composition of ecological communities often co-vary with ecosystem productivity. However, the relative importance of productivity, or resource abundance, versus the spatial distribution of resources in shaping those ecological patterns is not well understood, particularly for the bacterial communities that underlie most important ecosystem functions. Increasing ecosystem productivity in lakes has been shown to influence the composition and ecology of bacterial communities, but existing work has only evaluated the effect of increasing resource supply and not heterogeneity in how those resources are distributed. We quantified how bacterial communities varied with the trophic status of lakes and whether community responses differed in surface and deep habitats in response to heterogeneity in nutrient resources. Using ARISA fingerprinting, we found that bacterial communities were more abundant, richer, and more distinct among habitats as lake trophic state and vertical heterogeneity in nutrients increased, and that spatial resource variation produced habitat specific responses of bacteria in response to increased productivity. Furthermore, changes in communities in high nutrient lakes were not produced by turnover in community composition but from additional taxa augmenting core bacterial communities found in lower productivity lakes. These data suggests that bacterial community responses to nutrient enrichment in lakes vary spatially and are likely influenced disproportionately by rare taxa.

## Introduction

Ecosystem productivity is an important driver of the diversity and composition of ecological communities. Much attention has been given to understanding how communities change with increased productivity, due to the desire to understand how species and their threats are distributed globally [1] and the widespread increase in nutrient enrichment and primary productivity of many ecosystems [2]. Productive ecosystems often support high species richness [3], as evidenced by diversity hotspots in ecosystems such as marine upwelling zones [4] and tend to host distinct communities from low productivity ecosystems. Productivity is thought to promote changes in species richness and composition due to the increased energy available to support the coexistence of multiple species and trophic levels [5], [6], as well as by promoting shifts to species that dominate in productive environments. However, productivity is not always a good predictor of species richness [7], and the mechanisms behind observed richness and compositional changes in response to increased ecosystem productivity remain obscure [8].

Spatial or temporal heterogeneity in resource availability can also facilitate the coexistence of species in many environments [9], [10], and is commonly used to explain why species richness varies with ecosystem productivity [11]–[13]. Yet, the relative importance of resource availability and heterogeneity in influencing patterns of species richness and composition in productive ecosystems remains unclear for many ecological communities [8], [14]–[16], especially for prokaryotes. Bacteria are a fundamental component of food webs and provide the foundation for overall ecosystem functioning, yet we know relatively little about how bacterial communities respond to increases in productivity in most ecosystems [17]–[19]. In addition, bacteria have unique characteristics, such as metabolic flexibility and dormancy that might make their response to productivity and resource heterogeneity unique. In addition, bacteria can acquire new functional capacities through the exchange of genetic material [20], thus, taxonomic richness may be unresponsive to changes in productivity [21].

Lakes vary widely in productivity and the heterogeneity of resource distribution in response to variation in nutrient loading from human and watershed sources [15], [22]. Increased primary production, or trophic status, in lakes is associated with changes in species richness and composition of many ecological communities, including bacteria [23], [24]. The richness of macroorganisms often declines at richer trophic state, due to the dominance of phytoplankton that are less palatable or toxic to consumers [25], declines in littoral productivity [26], and changes to the physical and chemical characteristics of the lake environment [22]. Therefore, changes associated with increased lake trophic status often negatively impact diversity of lake communities, change their composition, and lead to the dominance of a few species through homogenization of food resources and reduction in habitat availability [15], [27]. Bacterial communities are known to shift in response to increased lake trophic status [28], but the fundamental mechanisms and importance of resource distribution in mediating those changes have not been fully explored.

Nutrient enrichment in lakes tends to magnify the vertical differences in physical and chemical characteristics such as nitrogen (N), phosphorus (P) and dissolved oxygen (DO) among lake strata [22]. However, existing studies of the response of bacterial communities to eutrophication have only evaluated the responses of surface communities or the integrated water column rather than habitat-specific responses [28], [29]. In stratified lakes, the surface layer (epilimnion) is typically warm, nutrient-poor, and productive, whereas the deep layer (hypolimnion) is cooler, richer in nutrients, and often low in dissolved oxygen (DO). These differences may be especially important when considering how the response of lake bacteria may differ from eukaryotic communities since vertical differences in physical and chemical conditions are known to structure bacterial communities in stratified lakes [30]–[32]. For example, low DO in the hypolimnion promotes the use of diverse energy pathways by bacteria such as denitrification and sulfate reduction that are not energetically advantageous in the oxic epilimnion, and therefore, could promote higher diversity of bacterial communities in the entire water column in response to increased trophic status. Therefore, bacterial communities are likely less similar among lake strata in high productivity (eutrophic) than in low productivity (oligotrophic) lakes.

In addition, although several studies have observed changes in bacterial communities with increased lake trophic status [24], [29], [33], few have identified which type of bacterial taxa are responsible for driving shifts in overall composition [34], [35]. For example, while there is increasing evidence that some taxa flourish in high productivity lakes [36], it is unclear whether taxonomic changes result from a complete turnover in the community [37], an increase in the relative abundance of a few key taxa [38], or the increased presence of previously rare or novel taxa that augment a core community of taxa present in low nutrient lakes. For example, a study that evaluated how dominant, common and rare taxa responded to another important disturbance in lakes, lake mixing, found that shifts in the bacterial community were driven by the increased dominance of a few taxa [38].

We evaluated how bacterial abundance, taxonomic richness, and composition changed among and within lakes along a gradient of increasing trophic state. In particular, we quantified the amount of variation in the bacterial response that was explained by trophic state, resource heterogeneity, and their combination. Second, we evaluated whether communities associated with different lake habitats (specifically the epi-, meta- and hypolimnion) responded differently to increased trophic status than communities assessed in the surface layer or integrated at the whole-lake scale. Finally, we evaluated which taxa were responsible for changes in community characteristics; specifically, we asked whether patterns were driven by turnover in the community or by additional taxa augmenting a core community present across all lakes. Thus, in this study we were able to address whether changes in the observed number of taxa and composition of bacterial communities followed the same patterns as eukaryotic communities in response to productivity in lakes and whether the distribution or abundance of resources was more important in shaping those patterns.

## Methods

**Figure 1:**
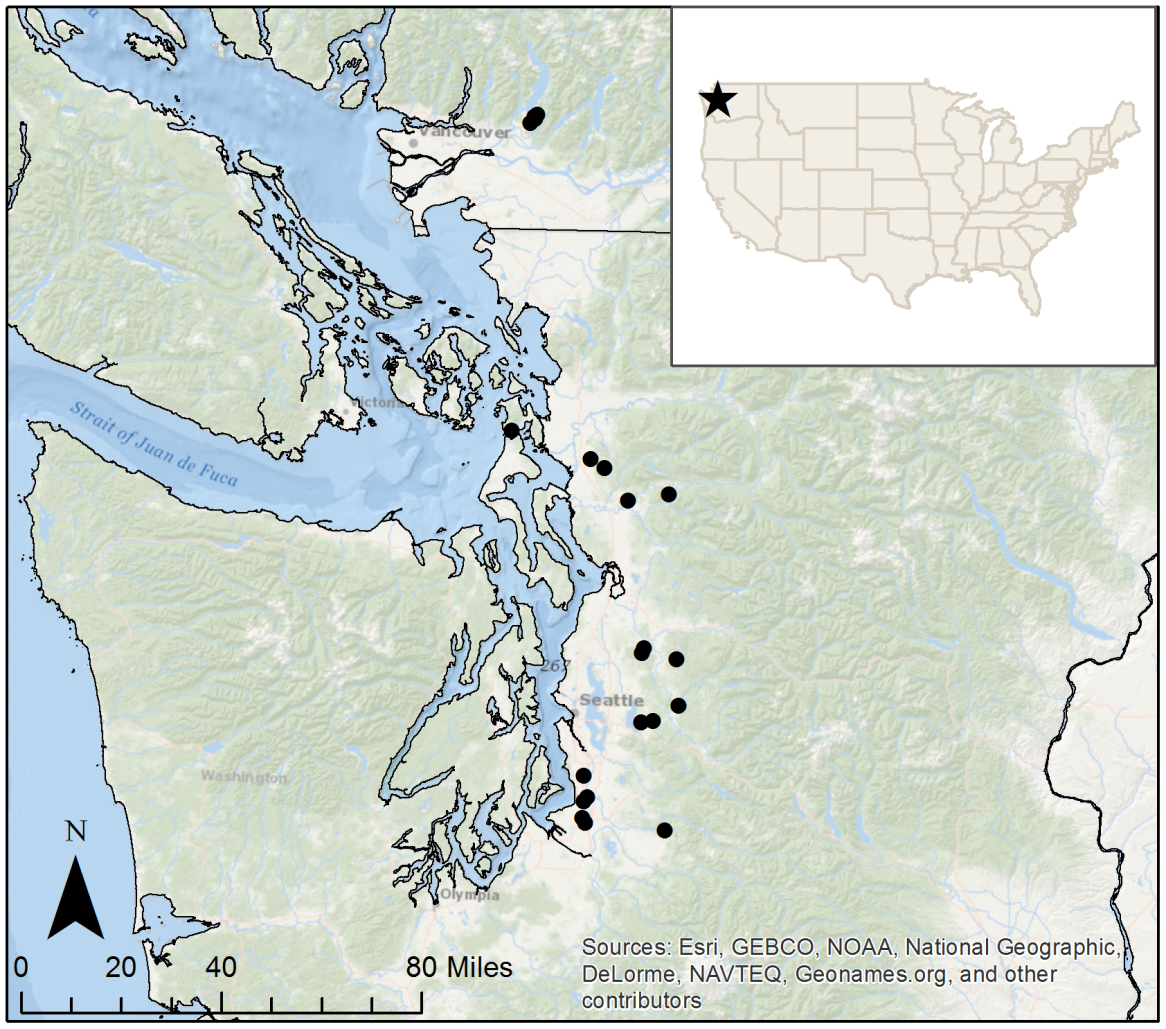
Map of study sites. Lakes included in the study were located in the Puget South Basin in Washington state, USA and British Columbia, Canada. Lakes are indicated by black points.

We sampled 21 lakes in the Puget Sound region of western Washington (USA) and southern British Columbia (Canada, Figure 1) that spanned a large gradient of anthropogenic nutrient loading and productivity [39]. We sampled during the summer-stratified period of July and August 2008. Therefore, our samples reflected the communities that had developed following two to three months of stratified conditions within the water column [31]. As previously described [39], the lakes included in this study were physically similar. Twenty of the 21 lakes were monomictic, and one lake was too shallow to develop thermal stratification. No permissions were required to access 17 of these lakes since they were accessible via a public boat launch. We obtained permission from the University of British Columbia to access the remaining four lakes, which were on the property of their Malcolm Knapp Research Forest. No endangered or protected species were involved in this research.

All bacterial community samples and measurements of lake environmental characteristics were collected over the deepest point in each lake. Water samples for nutrient and chlorophyll *a* analyses were collected from the epilimnion (surface), metalimnion (thermocline depth), and hypolimnion (within 3 m of the lake bottom) with a van Dorn bottle. Total N (TN) was determined using the perchloric acid digestion method [40] followed by analysis with automated colorimetry on a Lachat autoanalyzer (Lachat Instruments, Loveland, CO, USA). Total P (TP) concentration was determined colorimetrically after persulfate digestion and reaction with molybdate and stannous chloride [40]. Water samples for inorganic N and P determination were pre-filtered through a 0.2 mm Supor filters (Supor-200, Pall Gelman, East Hills, NY) and then analyzed colorimetrically using the same methods as above without a pre-digestion step. Chlorophyll *a* concentration was determined fluorometrically (Turner Designs, Sunnyvale, California) and used as a surrogate for algal community biomass. Temperature, dissolved oxygen (DO), and pH measurements were taken at 1-m depth intervals with a YSI sonde 6600 (YSI Integrated Systems & Services, Yellow Springs, OH, USA). Other physical lake data such as mean and maximum depth, lake area, and drainage area were obtained from the King County Water and Land Resources Division and the Washington Department of Ecology.

Two water samples for bacterial community analysis were collected from the epilimnion, metalimnion and hypolimnion of each lake with a Van Dorn Bottle. Two 300-mL samples were pooled and bacteria collected on 0.2-µm filters (Supor-200, Pall Gelman, East Hills, NY). Filters were frozen immediately and stored at −80°C until further processing. DNA was extracted from replicate filters using the Qiagen DNEasy Blood and Tissue Mini-kit (Qiagen, Valencia, CA). Samples for bacterial cell enumeration were preserved with 2% formalin, filtered onto a 0.2 µm black polycarbonate filter, stained with 4′, 6-diamidino-2-phenylindole (DAPI), and viewed with a Nikon Eclipse 80i digital microscope at 1000× magnification.

Bacterial community composition and observed richness were assessed using automated ribosomal intergenic spacer analysis [41]. ARISA generates fingerprints of the microbial community based on the length heterogeneity in the intergenic spacer region between the 16S and 23S rRNA genes, which varies among organisms. ARISA has similar limitations as other PCR-based fingerprinting approaches [41] and tends to only survey dominant taxa in a community, thus our assessment of bacterial community composition is really a comparison of the community of dominant taxa among lakes. However, ARISA has been shown to give a robust, high-resolution view of bacterial assemblages in aquatic ecosystems [42], [43], to generate results that are consistent with more high resolution techniques [42], [44], and can represent species-level taxonomic resolution (98–99% sequence similarity; [42]). The 16S-23S intergenic region was amplified using the polymerase chain reaction (PCR) from the total extracted DNA using 6-FAM-labelled universal 1406-F primer (5′ TGYACACACCGCCCGT-3′) and bacterial specific primer 23S-R(5′-GGGTTBCCCCATTCRG-3′) [41], [45]. PCRs were conducted on a Mastercycler gradient thermocycler (Eppendorf, New York). PCR products were pooled, quantified, and analyzed on a MegaBACE 1000 automated capillary sequencer (GE Healthcare Corporation, New Jersey). Operational taxonomic units (OTUs) were generated by binning ARISA fragments into successively larger length bins based on their size and eliminating fragments that were <150 and >1300 bp [42]. We used peak area to estimate relative abundance of OTUs in our samples [43], which we considered to be the ratio of the peak area of an OTU in a sample to the total peak area of the sample. We also converted the peak area matrix to presence-absence to assess the composition of bacterial communities in the ARISA profiles by occurrence patterns. We calculated observed richness from ARISA profiles by summing the number of OTUs observed in each sample, hereafter referred to as profile richness. We found no differences in bacterial community patterns using peak height vs. peak area.

### Statistical Analyses

**Figure 2:**
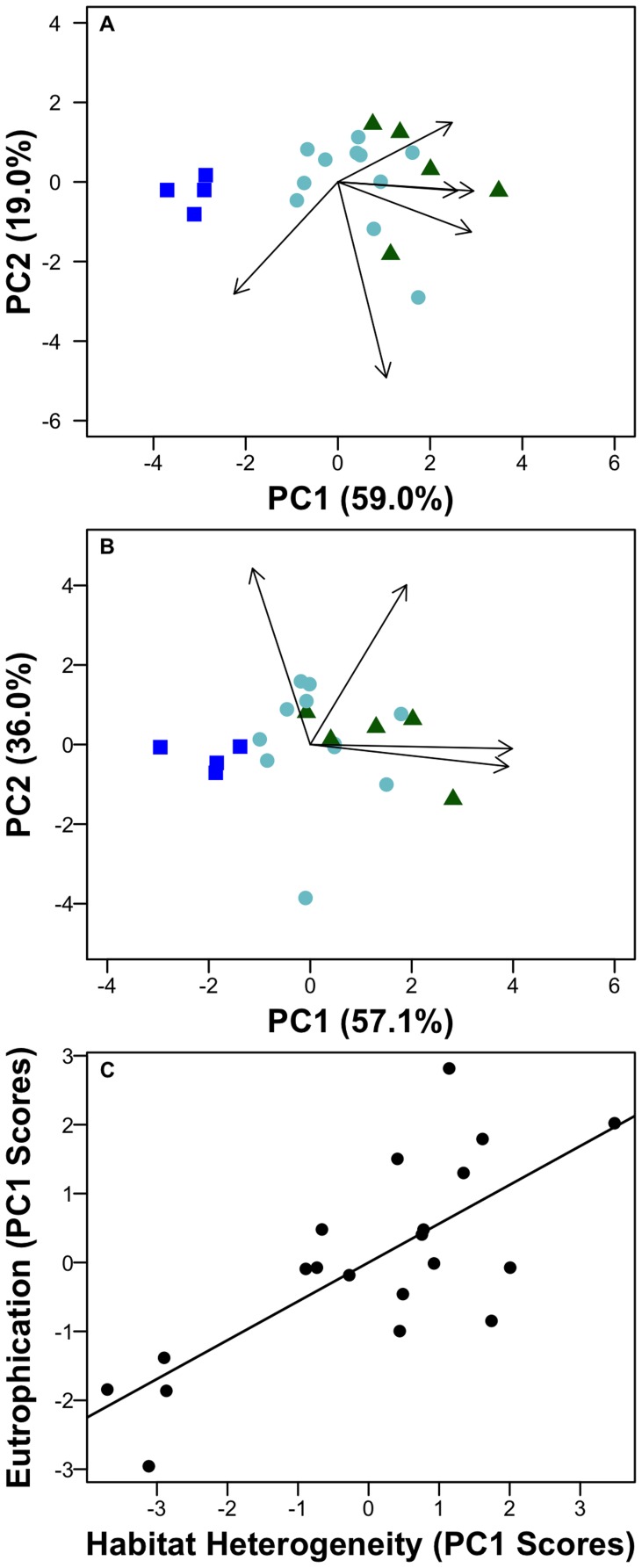
Principal component analyses (PCAs) showing environmental variation across lakes in this study. Panel A shows PCA results based on trophic state variables, panel B shows PCA results based on Heterogeneity variables (standard deviation among depths in variables) and panel C shows the correlation between the trophic state PC 1 scores and heterogeneity PC 1 scores (R^2^ = 0.57). Arrows show significant variables (p<0.05) and values in parentheses show percentages of total environmental variation among lakes explained by each axis. ‘EpiTN’ = epilimnetic TN, ‘EpiTP’ = epilimnetic TP, ‘HypoTP’ = Hypolimnetic TP, ‘HypoDO’ = hypolimnetic DO, and DO = epilimnetic DO. ‘.std’ indicates that the standard deviation of measurements of the specified variable among layers. Triangles = eutrophic, stars = mesotrophic, and squares = oligotrophic lakes.

We used a principal components analysis (PCA) to summarize physical and chemical variation related to trophic status among lakes. We found that lakes varied little in relevant physical characteristics (lake area and mean depth), and thus we used the first axis of the resulting PCA as a multivariate proxy for increasing lake trophic state (Figure 2A). Although we did not measure primary productivity directly, other studies have found good agreement between primary productivity and chlorophyll *a* and nutrient concentrations in lakes with similar concentrations as lakes in this study [46].

To quantify vertical heterogeneity in chemical and physical variables within each lake (e.g., TN, TP, temperature, and DO), we used the standard deviation of measurements among lake strata (Table S1). We then performed a PCA that included only the standard deviations of these physical and chemical variables to establish a gradient of resource heterogeneity among lakes.

To compare the influence of increasing trophic state (“trophic status”), depth variation in resource availability (“resource heterogeneity”), and their combination (trophic status and resource heterogeneity) on the bacterial community, we then took the scores from the first principal component (PC 1) of each PCA and regressed them against metrics of bacterial abundance, ARISA profile richness, and an index of community similarity among ARISA profiles (see below for description). The combined trophic status and resource heterogeneity model contained two predictor variables: PC 1 of the trophic status PCA, and PC1 of the resource heterogeneity PCA. All variables were transformed to meet the assumptions of normality prior to the PCA. We evaluated the support for each of the three candidate models describing the relationships between environmental conditions and the bacteria community attributes using Akaike’s Information Criteria adjusted for small sample sizes [47]. The model with the lowest AIC_c_ was considered the best model, and models within 2 AIC_c_ units of one another were considered to be equally good [47]. In addition, we calculated AIC weights (w_i_) for each individual model, which estimates a probability that model *i* is the best model given the set of models we considered. Finally, to evaluate overall importance of the individual variables, trophic status and resource heterogeneity, we calculated w_i_ for each term across the three models we compared (Table 1).

**Table 1:** Comparison of models evaluating the effects of trophic state, heterogeneity, and their combination on bacterial communities.

	Model	n	k	R^2^	AIC_c_	ΔAIC_c_	w_i_ *	w_i_ of T+	w_i_ of H++
**ABUNDANCE**									
	Trophic State	21	3	0.46	612.2	9.1	0.01	0.34	0.83
	Heterogeneity	21	3	0.66	603.1	0.0	0.50		
	PC1 T+PC1 H	21	4	0.69	603.3	0.0	0.33		
**RICHNESS**									
	Trophic State	17	3	0.30	129.3	2.9	0.16	0.33	0.84
	Heterogeneity	17	3	0.41	126.4	0.0	0.67		
	PC1 T+PC1 H	17	4	0.40	129.2	2.8	0.17		
**DISSIMILARITY**									
	Trophic State	17	3	0.12	−23.8	0.9	0.35	0.46	0.65
	Heterogeneity	17	3	0.17	−24.7	0.0	0.54		
	PC1 T+PC1 H	17	4	0.12	−21.5	3.1	0.11		
**WIDESPREAD**									
**TAXA**	Trophic State	18	3	0.29	−36.9	1.5	0.26	0.44	0.74
	Heterogeneity	18	3	0.35	−38.4	0.0	0.56		
	PC1 T+PC1 H	18	4	0.35	−36.1	2.3	0.18		

* AIC_c_ weight,

+ Trophic State,

++ Heterogeneity.

Relationship of trophic state, heterogeneity, and their combination with abundance, richness, dissimilarity of bacterial communities among lake habitats (“Dissimilarity”), and the proportional abundance of common taxa. ‘n’ = sample size and ‘k’ = number of parameters in each model. ‘w_i_’ refers to the AIC_c_ weight calculated for each model and the weights for the individual terms ‘T’ and ‘H’ across all models.

Bacterial community similarity among samples was assessed using Sorensen’s coefficient for occurrence data [48] and the Chao-Sorensen abundance estimator for relative abundance data [49]. We assessed the overall similarity of communities within a lake by using an average dissimilarity value among ARISA profiles from all two-layer comparisons. We used a constrained analysis of principal coordinates (CAP) to evaluate if changes in community composition were associated with increasing trophic state [50] since it allowed us to use the Chao-Sorensen similarity index.

Finally, we investigated whether changes in the bacterial community with increased lake trophic status were realized by shifts in “widespread” or “narrowly distributed” taxa. We assessed how the relative contribution of widespread OTUs (taxa observed in the majority of lakes) changed with trophic state and habitat heterogeneity. We considered widespread taxa to be those that were observed in 90% of lakes in our study (but see Table S2 for evaluation of different thresholds). We then assessed whether the occurrence and relative abundance of these taxa changed across the lake trophic gradient and with increasing heterogeneity (i.e., PC1 of trophic status and resource heterogeneity PCAs). All analyses were done in R Version 2.14.0 [51] using the vegan package [52].

## Results

### Lake Characteristics

Productivity-related variables such as TP, TN, and chlorophyll a explained a substantial portion of the environmental variation among lakes in this study (59%; Figure 2, Table S1). Epilimnetic TP concentrations ranged from 4.6 µ g L^−1^ to over 30 µg L^−1^, and chlorophyll *a* ranged from 0.23 to 10.2 µg L^−1^, thus the lakes ranged from oligotrophic to eutrophic [22]. Environmental conditions did not change similarly in each layer with increased trophic state; for example, the epilimnion was less variable among lakes than either the metalimnion or hypolimnion in most environmental characteristics (Table S3). As a result, conditions within the water column were more heterogeneous as trophic state increased (R^2^ = 0.57, Figure 2C). We observed the most significant differences in TN and TP concentrations among layers as trophic state increased (Figures 2B and C). TN and TP were correlated with the availability of NH_4_ (r = 0.71) and PO_4_ (r = 0.86), respectively. Thus, nutrient availability was variable within the water column. Finally, the percent change in nutrient concentrations was greater in the hypolimnion than in either the epi- or metalimnion (Table S3).

### Did Lake Bacterial Communities Shift in Response to Increasing Trophic State?

**Figure 3:**
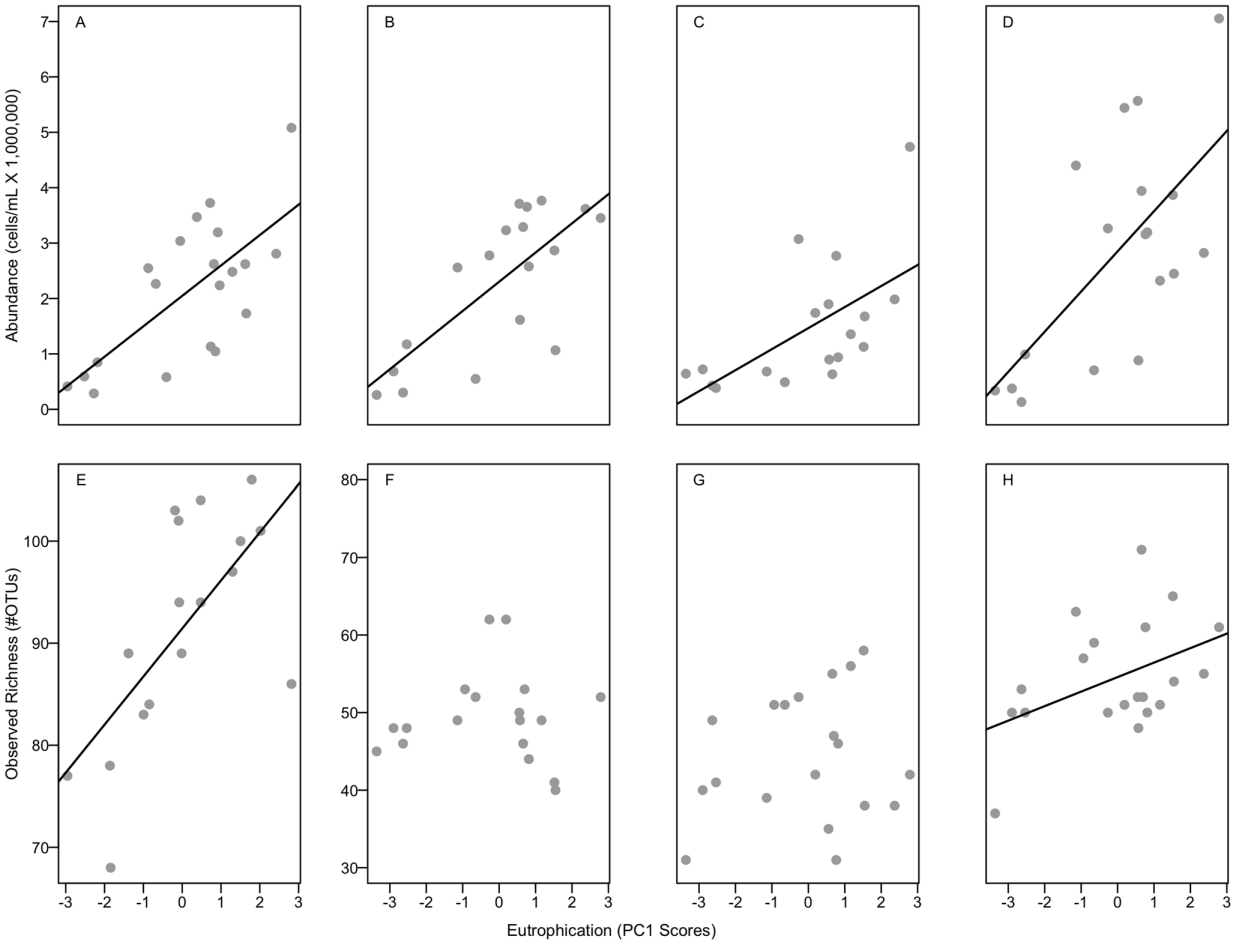
Relationships of increasing trophic state (PC 1 scores) with abundance and richness. A) Whole-lake average abundance (R^2^ = 0.46, p<0.001), B) Epilimnetic abundance (R^2^ = 0.52, p<0.001), C) Metalimnetic abundance (R^2^ = 0.33, p = 0.007), D) Hypolimnetic abundance (R^2^ = 0.40, p = 0.003), E) Whole-lake average richness (R^2^ = 0.30, p = 0.003), F) Epilimnetic richness (R^2^ = −0.06, p = 0.98), G) Metalimnetic richness (R^2^ = −0.03, p = 0.49), and H) Hypolimnetic richness (R^2^ = 0.15, p = 0.05).

Bacterial communities at the whole-lake scale shifted significantly in association with increasing lake trophic state (Figure 3, Figure S1). Average bacterial abundance (R^2^ = 0.46, Table 1, Figure 2A) and ARISA profile richness increased linearly with our proxy for lake eutrophication (R^2^ = 0.30, Table 1, Figures 3A & E) and ranged from 66 to 106 OTUs per lake. Our CAP model showed that bacterial community composition shifted with increased lake trophic status and shifts were strongly associated with increasing chlorophyll *a* (r = 0.99) and epilimnetic TN (r = 0.76) concentration (Figure S1). The CAP model explained 29% of the total variation in community composition among lakes. The first CAP axis captured the majority of that explained variation (71.4%), indicating that community composition changed in response to increased lake trophic state.

### Did the Responses of the Bacterial Community Vary among Habitats in Lakes?

Bacterial communities associated with surface and deep habitats displayed different patterns of abundance, and the richness and composition of ARISA profiles changed significantly as lake trophic state increased (Figure 3, Figure S1). Average bacterial abundance increased with trophic state (R^2^ = 0.46), was highest in the metalimnion (ANOVA; F = 5.6, p = 0.006), but increased significantly in all layers across the trophic gradient (Figure 3). The richness of ARISA profiles also varied significantly among layers (ANOVA, F = 10.6, p<0.001), but only increased notably in the hypolimnion in response to trophic state (R^2^ = 0.15, Figure 3H). Profile richness was highest on average in the hypolimnion (55±7 SD), which also had the largest range of observed richness, ranging from 37 OTUs in Gwendoline Lake, an oligotrophic lake, to 71 OTUs found in more nutrient-rich Geneva Lake. Therefore, increases in the profile richness in the hypolimnion accounted for the increases we observed in overall lake richness (R^2^ = 0.39).

**Figure 4:**
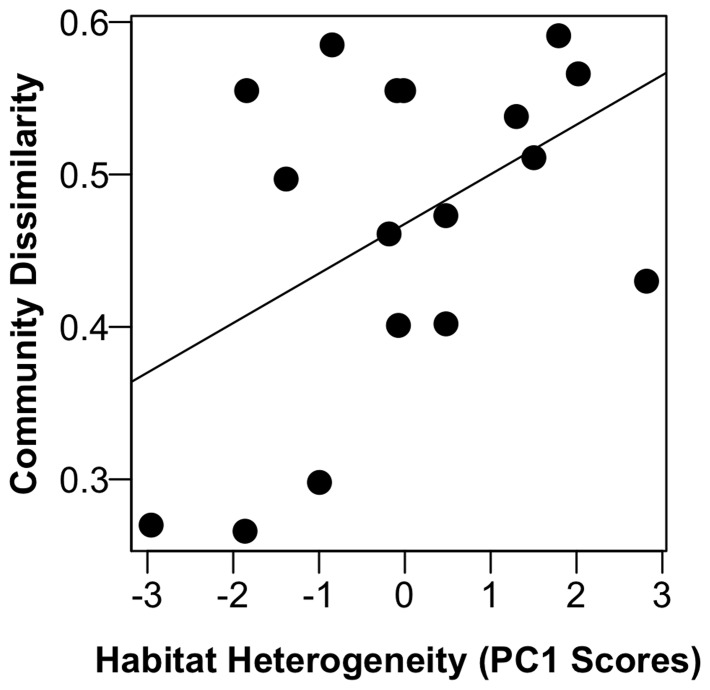
Bacterial communities were less similar among lake strata as chemical heterogeneity increased (R^2^ = 0.17, p = 0.05). Similarity of communities was based on the average Chao Sorensen Dissimilarity from comparisons of relative abundance of taxa among three depth strata.

When all lake communities were considered together, there were significant, but small, compositional differences among epi-, meta- and hypolimnetic communities (ANOSIM, R = 0.16, p = 0.001), and surface and deep communities shared the fewest taxa (data not shown). Furthermore, surface communities were significantly less variable than deep communities across the trophic gradient (Homogeneity of dispersion, p<0.001), and surface and deep communities within a given lake tended to become less similar to one another as trophic state increased (R^2^ = 0.12). However, heterogeneity in nutrient concentrations among strata explained slightly more of that variation than trophic state alone (R^2^ = 0.17, Figure 4, Table 1).

**Figure 5:**
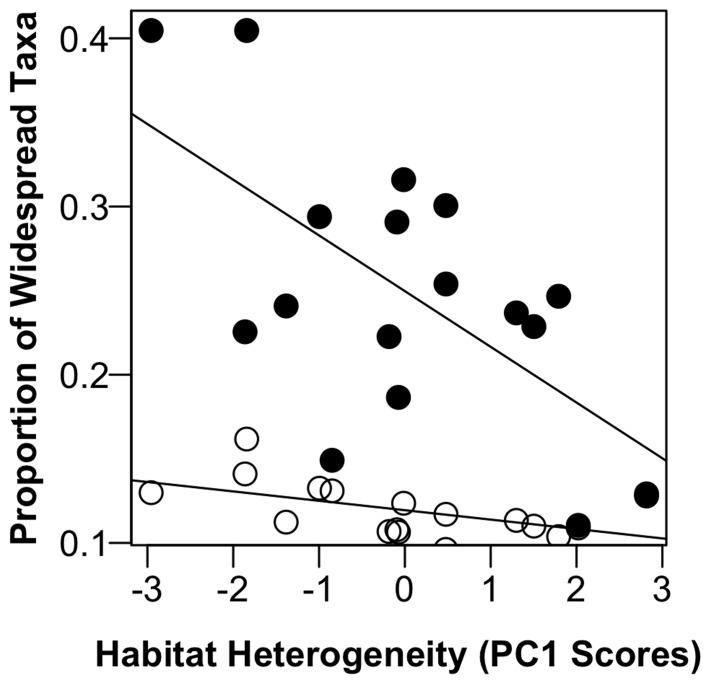
Relationship of habitat heterogeneity with the proportional representation of widespread taxa in each lake. Heterogeneity was measured as the PC 1 scores of ‘heterogeneity’ model. Filled circles show the total proportion of relative abundance made up by widespread taxa (R^2^ = 0.35, p = 0.007) and open circles show widespread taxa as a proportion of the total number of taxa observed (R^2^ = 0.23, p = 0.03).

In all cases, the heterogeneity model or the trophic status plus heterogeneity model explained more variation in bacterial communities among lakes than the trophic status model alone (Table 1). We found that greater vertical heterogeneity of nutrient availability (Figure 2) was strongly related to increased abundance, ARISA profile richness, and decreasing similarity of communities among lake strata (Table 1, Figure 4). Total abundance and observed richness were both more strongly related to increases in the heterogeneity of P and N than increases in their concentrations alone or to differences in temperature and DO among strata, which other studies have shown to be associated with heterogeneity in bacterial community composition (Table S1; [31], [32]). Therefore, although we saw an increase in observed richness with increased trophic state (R^2^ = 0.30), observed richness was more closely linked to greater heterogeneity of nutrients within the water column (R^2^ = 0.41). Overall, the AIC_c_ shows that the heterogeneity model had the most support, and that, in fact, the heterogeneity term had the most weight across models (Table 1). Thus, as measured here, bacterial communities exhibited habitat-specific responses to lake eutrophication, and spatial variation in resource availability often influenced bacterial community composition more than simple increases in nutrient concentration and productivity (Figures 4 and 5).

### Which Taxa Accounted for changes in Community Composition with Eutrophication?

We observed a total of 221 OTUs across all lakes and found that some of those taxa were widespread among lakes. We observed that while a core community of 11 OTUs was present and detected using ARISA in ∼90% of lakes in this study (“widespread taxa”), and while still present across lakes, made up a decreasing proportion of both the number (R^2^ = 0.23) and relative abundance of taxa in lakes as lake trophic state increased (R^2^ = 0.35, Figure 5). Specifically, while these widespread taxa comprised 35–40% of the relative abundance of the community in the more homogenous oligotrophic lakes, they were less than 15% in the more heterogeneous eutrophic lakes. These trends were robust to using different thresholds to define “widespread” (e.g., present in 70–90% of lakes), and strengthened as we considered thresholds up to 90% (Table S2). We only observed four OTUs that were present in >90% of lakes, which likely were ubiquitous taxa that would be present regardless of lake trophic state [36]. In addition, we found that the increasing representation of previously low abundance or new taxa in response to trophic status was explained most by increasing heterogeneity among habitats (Table 1). Although the AIC_c_ values for all three models were very close, the AIC value and variable w_i_’s suggest that heterogeneity explained the declining contribution of the widespread OTUs we observed to the overall lake community (Table 1). This suggests that either novel, previously low abundance, (i.e., below the detection limit of ARISA), or dormant taxa [35] increased in their relative importance as nutrient status of lakes increased, and that chemical heterogeneity in the lake environment most likely facilitated the increased prevalence of these taxa in the lake community.

## Discussion

We found that the abundance and richness of bacterial communities increased as lake nutrient status increased, in parallel to what has been reported in other studies [28], [33], [45]. However, we found even stronger relationships between the overall abundance and number of OTUs observed and the spatial heterogeneity in nutrient conditions among lake layers as trophic status increased. Additionally, because environmental conditions among lake strata diverged and communities associated with these habitats responded differently to lake trophic status (Figure 3), bacterial communities as measured by ARISA fingerprinting were much less similar among lake strata as lake trophic state increased (Figure 4, Table S3). Furthermore, our analyses demonstrated that a core bacterial community of dominant bacteria did not change systematically in high nutrient lakes but rather that increasing habitat heterogeneity, specifically due to large changes in conditions in the deepest layer of lakes, provided additional habitat for previously low abundance or absent taxa that became detectable in more eutrophic conditions. Our study shows that the response of bacterial communities to increased productivity in lakes may differ from that of other lake organisms as a result of spatial heterogeneity in resources specifically affecting the ecology of bacteria and supports the idea that the recruitment of rare or dormant bacterial taxa in lake communities may facilitate much of this response (e.g., [35]).

### Effects of Trophic State on Lake Bacterial Communities

We observed significant increases in bacterial abundance and changes in the richness and composition of ARISA profiles at the whole-lake scale in response to increases in lake trophic status. As expected, abundance was positively related to increases in nutrient availability (TN and TP) and chlorophyll *a* (Table 1) across the entire range of ecosystem productivity we observed. High abundances of bacteria are often correlated with high rates of bacterial productivity [46], which can be nutrient-limited in oligotrophic lakes. We also observed increased ARISA profile richness as trophic state increased (Figure 3E), suggesting that more taxa coexisted as dominant types (and thus able to be detected by ARISA) as resource availability increased. As has been observed for macroorganisms in lakes [23], other studies have shown lake bacterial richness to exhibit a range of linear and unimodal responses to productivity depending on the spatial and taxonomic resolution of the study [17], [18], [29]. We likely observed a linear increase in observed richness since our lakes represented a relatively modest gradient in nutrient loading and chlorophyll *a* compared to the global distribution of lake productivity [22], thus, we may have only captured the initial upward slope of a unimodal response across the global range of lake trophic states. Alternatively, is also possible that we observed a consistently increasing number of taxa rather than a unimodal trend due an effect of increased sampling; in other words, because we took discrete samples from each lake habitat and ran a separate ARISA analysis on each sample rather than on one integrated sample from each lake, we were able to detect more taxa than previous studies [23], [29].

### Bacterial Community Response to Resource Heterogeneity

Bacterial community shifts were most strongly associated with changes in the heterogeneity of the lake environment in all cases (Table 1). Specifically, we observed more OTUs and decreased similarity among bacterial communities in surface and deep habitats in response to heterogeneity in N and P concentrations (Figures 2B and 3). Environmental conditions within lakes changed to different degrees among the epi-, meta- and hypolimnion (Table S3), which translated to habitat specific responses of bacterial communities in each layer across lakes (Figure 3). For instance, although bacterial abundance increased in all layers, we only observed increases in richness in the hypolimnion with increased trophic status (Figure 3H), which also had the largest range in nutrient concentrations across the trophic gradient (Figure S1). Interestingly, communities in the surface layer varied less across the trophic gradient than communities in the hypolimnion, suggesting that studies that evaluate trends in bacterial communities in response to eutrophication miss an important aspect of the bacterial response.

While our study design does not allow us to definitely conclude whether patterns in bacterial communities were related more to increased trophic state or to habitat heterogeneity because the two were correlated (Figure 2C), we found strong support for our heterogeneity model (Table 1) and patterns suggesting that both were important for bacterial communities. For instance, we observed lower richness (66 OTUs) and higher similarity of communities among lake strata (Sorensen similarity = 0.86) in the only lake in our study that did not stratify into discrete habitats. Although this lake had similar nutrient concentrations as eutrophic lakes, environmental conditions were more homogeneous within the water column in this lake suggesting that heterogeneity in resource availability influenced bacterial richness more than nutrient concentration alone in all lakes in our study.

While changes in bacterial communities in response to increasing lake trophic status have been widely observed [28], [29], [45] as have differences in communities among lake strata [30], [31], [53] previous studies have not linked these observations to evaluate the combined effects of lake resource availability and resource heterogeneity on the richness and composition of lake bacterial communities. Thus, while similar communities may inhabit eutrophic lakes [36], communities become increasingly distinct from one another as surface and deep habitat conditions diverge and biotic interactions change [54]. Furthermore, these findings contrast with studies of other lake organisms, such as zooplankton and fish, which find increased productivity reduces diversity as a result of homogenization of food resources and loss of habitat [15], [26], [27]. Thus, our study demonstrates that the type of heterogeneity that influences communities varies among macro- and microorganisms and that the response to a large-scale environmental change is expressed differently among habitats within ecosystems.

In addition, our results suggest that changes in bacterial communities with increased trophic status may be more strongly related to vertical differences in nutrient concentrations than dissolved oxygen, temperature, and light, which are typically thought to structure differences among communities in different strata within lakes [24], [30], [31], [53]. We tested this by regressing axis 1 and 2 of our environmental PCA (Figure S1), which represented differences among layers in nutrient concentrations and DO/temperature, respectively. We found that in all cases axis 2 (variation in DO and temperature) did not have a strong relationship with any bacterial response measure. Few studies have tested for this and those that have, have often focused on a single lake or small set of lakes [30], [53]. Thus, these results suggest that increased vertical differences in nutrient concentrations may be more important in structuring the bacterial community than vertical differences in DO and temperature as the trophic state of lakes increases.

### Were Widespread or Narrowly Distributed Taxa Responsible for Shifts in Community Composition?

We found that habitat heterogeneity played an important role in shaping the bacterial response to increasing lake trophic status through enabling the increased contribution of new or previously low abundance taxa in lake communities. Furthermore, we found that increased resource heterogeneity simultaneously allowed for the retention of a core group of taxa that were widespread among lakes while also providing new habitat and resources for these previously unobserved or low abundance taxa (Figure 5). Thus, our results support the general notion of the importance of rare taxa in microbial communities [35], [55] and suggest that bacterially-relevant habitat heterogeneity may be an important mechanism driving the bacterial response to increased lake ecosystem productivity. Additionally, this pattern may be common to many types of planktonic communities [34]. For instance, a study of the response of a lake phytoplankton community to eutrophication and recovery found that there were phytoplankton that were consistently present through time, but that temporally rare taxa were most responsive to changes in lake nutrient status and drove changes in community composition [56].

While the use of ARISA allowed us to screen the bacterial community in a large number of lakes and has been shown to have species-level taxonomic resolution, as with so many methods used to sample bacterial communities, there are limitations associated with using this approach [57]. For example, ARISA underestimates the total richness of the bacterial community and has biases such as preferential amplification of abundant organisms [41]. For example, many of the additional taxa that we observed in more eutrophic lakes could have been present at low abundances in oligotrophic lakes, and therefore below the detection limit of ARISA. However, increased detection of these taxa in eutrophic lakes suggests that they are at higher abundances and thus may be more functionally important in those communities. In addition, other studies have shown that ARISA captures similar patterns in diversity among communities as more high-resolution techniques such as clone libraries [58]. Further, although sequencing and clone library techniques would have allowed us to identify specific taxa, the higher costs associated with those techniques would have limited our ability to sample the entire trophic gradient in our study. Finally, our results are comparable to other studies using similar techniques [24], [33], [35], and recent studies of bacterial responses to nutrient additions that have used high throughput sequencing techniques suggest that using a more thorough sampling approach likely would not likely reveal a different trend in how richness and composition respond to increased ecosystem productivity (e.g., [19], [44]). Thus, the fact that we observed such striking trends using this approach suggests that higher resolution sampling would have only strengthened observed patterns.

In summary, we showed that bacterial community composition changed and was richer and more heterogeneous within lakes as trophic status increased. In contrast to trends in macroorganisms whose diversity is often negatively associated with increases in lake productivity [15], [23], [27], we showed that the high degree of heterogeneity in bacterial resources in eutrophic lakes promoted higher richness as a result of differentiation of bacterial taxa among lake habitats. We found that eutrophication alters the drivers of bacterial community differences within lakes from physical and redox related variables to changes in nutrient availability. Furthermore, our results suggest that rare or dormant taxa may be most responsible for changes in bacterial communities with increased lake trophic state [35], [59]. This “seed bank” of taxa has increasingly been recognized to be important in responding to changes in many types of ecosystems [60], and understanding the role of rare and dormant taxa an important frontier for understanding the processes that regulate how microbial communities respond to ecosystem change in general.

## Supporting Information

Figure S1 **Constrained analysis of principal coordinates (CAP) of bacterial community composition with environmental variables.**
(DOCX)

Table S1 **Results of eutrophication and heterogeneity Principal Components Analyses (PCAs).**
(DOCX)

Table S2 **Model results from comparing effects of trophic state, heterogeneity, and the combination of the two (T+H) on the relative abundance of widespread taxa in lakes.**
(DOCX)

Table S3 **Means and variation of key environmental variables among and within lakes.**
(DOCX)
