# Supplementary material for: Resource Availability and Spatial Heterogeneity Control Bacterial Community Response to Nutrient Enrichment in Lakes
Source: PLoS One. 2014 Jan 28;9(1):e86991. doi: 10.1371/journal.pone.0086991 (PMC3904960; doi:10.1371/journal.pone.0086991)
Supplement: Figure S1 — Constrained analysis of principal coordinates (CAP) of bacterial community composition with environmental variables. (DOCX) [file pone.0086991.s001.docx]

**Figure S1. Constrained analysis of principal coordinates (CAP) of bacterial community composition with environmental variables.** CAP explains 29% of variation in community composition among lakes. Arrows show variables significantly related to bacterial community composition (p < 0.05). Values in parentheses show percentages of total variation in bacterial community composition explained by each axis. ‘EpiTN’ = epilimnetic TN, ‘EpiTP’ = epilimnetic TP, and DO = epilimnetic DO. Triangles = eutrophic, stars = mesotrophic, and squares = oligotrophic lakes.
