# Supplementary material for: Resource Availability and Spatial Heterogeneity Control Bacterial Community Response to Nutrient Enrichment in Lakes
Source: PLoS One. 2014 Jan 28;9(1):e86991. doi: 10.1371/journal.pone.0086991 (PMC3904960; doi:10.1371/journal.pone.0086991)
Supplement: Table S1 — Results of eutrophication and heterogeneity Principal Components Analyses (PCAs). (DOCX) [file pone.0086991.s002.docx]

**Table S1.** **Results of eutrophication and heterogeneity** **Principal Components Analyses (PCAs).** Correlation coefficients of significant variables are shown in parentheses. Variable names followed by “.std” signify the standard deviation of measurements of those variables among lake strata. “PC” = principal component.
