# Supplementary material for: Resource Availability and Spatial Heterogeneity Control Bacterial Community Response to Nutrient Enrichment in Lakes
Source: PLoS One. 2014 Jan 28;9(1):e86991. doi: 10.1371/journal.pone.0086991 (PMC3904960; doi:10.1371/journal.pone.0086991)
Supplement: Table S2 — Model results from comparing effects of trophic state, heterogeneity, and the combination of the two (T+H) on the relative abundance of widespread taxa in lakes. (DOCX) [file pone.0086991.s003.docx]

**Table S2.**  **Model results from comparing effects of trophic state, heterogeneity, and the combination of the two (T+H) on the relative abundance of widespread taxa in lakes.** Table shows results of model when using different threshold values for defining widespread (percent of lakes present). ‘k’ = number of parameters and ‘n’ = sample size.
