# Supplementary material for: Resource Availability and Spatial Heterogeneity Control Bacterial Community Response to Nutrient Enrichment in Lakes
Source: PLoS One. 2014 Jan 28;9(1):e86991. doi: 10.1371/journal.pone.0086991 (PMC3904960; doi:10.1371/journal.pone.0086991)
Supplement: Table S3 — Means and variation of key environmental variables among and within lakes. (DOCX) [file pone.0086991.s004.docx]

**Table S3**. **Means and variation of key environmental variables among and within lakes.** S.D. = standard deviation, C.V. = coefficient of variation.
